# Novel Homozygous *LAMC3* Frameshift Variant Associated with Confluent Leukoencephalopathy and Low-Grade Tectal Glioneuronal Tumor: Expanding the Phenotypic Spectrum with Bioinformatic Characterization

**DOI:** 10.3390/life16091485

**Published:** 2026-09-05

**Authors:** Serdar Bozlak, Cuneyd Yavas, Halil Ibrahim Yilmaz, Ozan Sonmez, Peren Perk, Tuna Eren Esen, Duygu Cetinkaya, Tunay Dogan, Sirin Bozlak

**Affiliations:** 1 Department of Molecular Biology and Genetics, Faculty of Engineering and Natural Sciences, Biruni University, Istanbul 34460, Turkey; 2 Department of Pediatric Genetics, University of Health Sciences Basaksehir Cam and Sakura City Hospital, Istanbul 34480, Turkey; 3 Department of Medical Genetics, University of Health Sciences Basaksehir Cam and Sakura City Hospital, Istanbul 34480, Turkey; 4 Nevagen Genetic Diagnosis Center, Istanbul 34100, Turkey; 5 Department of Medical Laboratory Techniques, Vocational School of Health Care Services, Istinye University, Istanbul 34396, Turkey; 6 Vocational School of Health Services, Altınbaş University, Istanbul 34147, Turkey

**Keywords:** *LAMC3*, laminin gamma-3, leukoencephalopathy, white matter disease, tectal glioma, cortical malformation, novel variant, phenotypic expansion, whole-exome sequencing, autosomal recessive

## Abstract

Background: Biallelic loss-of-function variants in *LAMC3*, encoding laminin gamma-3, cause occipital cortical malformation (OMIM#614115). White matter disease and intracranial neoplasia have not been reported in this spectrum. We report a novel homozygous *LAMC3* frameshift variant, expanding its phenotypic and molecular spectrum. Methods: Two adolescent siblings from a consanguineous Turkish family underwent whole-exome sequencing, with segregation confirmed by NGS/IGV and classification per ACMG/AMP criteria. In silico analyses included multiple sequence alignment, AlphaFold modeling of wild-type and mutant proteins, and docking against nidogen-1 (NID1). Results: Both siblings had a novel homozygous *LAMC3* variant frameshift variant (NM_006059.4: c.1852_1882del; p.(Pro618Serfs*5)), classified as pathogenic (PVS1, PM2, PP3, PP1) with full cosegregation. Proband II.III, a 17-year-old female, developed postoperative epilepsy after resection of a tectal low-grade glioneuronal tumor harboring a somatic *KRAS* (NM_004985.3) p.(Gln61Lys) variant (VAF 42.9%), with periventricular white matter gliosis. Proband II.IV, a 15-year-old male, presented with confluent leukoencephalopathy, occipital pachygyria, parietal polymicrogyria, and subcortical band heterotopia, illustrating striking intrafamilial discordance. Conclusions: Docking analysis revealed that the cleavage removes the C-terminal nidogen-binding region, eliminates the predicted wild-type interface (residues 906–1029), and shifts the binding to an unnatural N-terminal surface. This finding is a hypothesis-generating result consistent with loss of function. This study expands the *LAMC3* phenotype to include leukoencephalopathy and reports a co-occurring low-grade tectal glioneuronal tumor as a novel, single-case observation, supporting inclusion of *LAMC3* in the differential diagnosis of pediatric leukoencephalopathies, particularly with consanguinity.

## 1. Introduction

Malformations of cortical development (MCDs) are a heterogeneous group of disorders characterized by abnormal cerebral cortex formation, most commonly presenting with epilepsy and neurodevelopmental delay [1,2]. Recessive *LAMC3* gene variants cause occipital cortical malformations (OMIM #614115), a rare autosomal recessive condition first described by [3] in consanguineous Turkish families presenting with complex bilateral occipital gyration abnormalities. *LAMC3* encodes the laminin gamma-3 chain, a critical extracellular matrix protein involved in cell differentiation, migration, and adhesion, playing an essential role in cortical organization and gyration [1,3].

*LAMC3*-related disease is exceptionally rare, with fewer than fifteen unrelated families reported in the literature to date. The emerging literature has progressively expanded the phenotypic spectrum beyond its originally defined occipital boundaries, encompassing frontal polymicrogyria, periventricular nodular heterotopia, temporal cobblestone malformation, and seizures with entirely normal MRI. However, white matter disease as a primary neuroradiological feature and the co-occurrence of intracranial neoplasia have not been previously described in association with *LAMC3* variants [4,5,6].

In the present study, we report two adolescent siblings from a consanguineous Turkish family harboring a novel homozygous *LAMC3* frameshift variant (c.1852_1882del; p.(Pro618Serfs*5)) identified by whole-exome sequencing. Both siblings exhibited features not previously associated with *LAMC3*-related disease, namely confluent leukoencephalopathy as the primary neuroradiological finding in one sibling and a low-grade tectal glioneuronal tumor in the other. We aimed to define their clinical, neuroradiological, and molecular characteristics, to establish the pathogenicity of the variant through familial segregation and ACMG/AMP classification, and to characterize its structural consequences in silico by conservation analysis, AlphaFold modeling of the wild-type and mutant proteins, and docking against nidogen-1 (NID1). In doing so, we expand the phenotypic and molecular spectrum of *LAMC3*-related disease and highlight *LAMC3* in the differential diagnosis of pediatric leukoencephalopathies of undetermined etiology.

## 2. Methods

### 2.1. Genetic Analysis

Written informed consent was obtained from the patients, their parents, and all available family members for clinical evaluation, genetic testing, and publication. The study was approved by the Başakşehir Çam and Sakura City Hospital Non-Interventional Clinical Research Ethics Committee (Approval No: KAEK/11.03.2026.89; Date: 11 March 2026) and conducted in accordance with the principles of the Declaration of Helsinki. Detailed clinical histories and neurological assessments were completed for all available family members.

Genomic DNA was extracted from peripheral blood leukocytes of the affected siblings and available family members using the QIAGEN DNeasy Blood DNA Extraction Kit (Qiagen, Hilden, Germany). Whole-exome sequencing was performed on the index patients; exonic and flanking splice-site regions were enriched with the QIAseq xHYB Actionable Exome (Qiagen, Hilden, Germany) and sequenced on the Element AVITI System (Element Biosciences, San Diego, CA, USA) with 100 bp paired-end reads at a mean on-target depth of approximately 100×. Reads were quality-trimmed, aligned to the human reference genome (GRCh37/hg19) using BWA-MEM, and processed for duplicate marking and variant calling with a GATK-based pipeline. Variants were annotated with ANNOVAR/VEP (v116) and filtered against population databases (gnomAD, 1000 Genomes), excluding those with a minor allele frequency above 0.5%. Given the parental consanguinity and the autosomal recessive inheritance of *LAMC3*-related disease, rare homozygous protein-altering variants were prioritized. The candidate variant in *LAMC3* (NM_006059.4) was evaluated using in silico predictors within dbNSFP and classified according to the ACMG/AMP guidelines [7]. Segregation of the c.1852_1882del variant was assessed in all available family members by next-generation sequencing, and read alignments were reviewed in the Integrative Genomics Viewer (IGV) to confirm zygosity in each individual. A structured literature search was performed in PubMed and Google Scholar to identify all previously reported LAMC3-related cases, using the search terms “LAMC3” combined with “cortical malformation,” “polymicrogyria,” “pachygyria,” or “epilepsy,” restricted to publications in English through June 2026. Case reports, case series, and original research articles reporting individual-level phenotypic and genotypic data were included; review articles without new patient-level data, conference abstracts without full-text availability, and studies lacking molecular confirmation of LAMC3 variants were excluded. When multiple publications described overlapping cohorts or the same family, the most complete and recent report was used as the primary source, cross-referenced against earlier reports to avoid case duplication in Table 1.

### 2.2. Bioinformatic Analysis

The canonical protein sequences of human laminin subunit gamma-3 (LAMC3; UniProt Q9Y6N6; 1575 aa) and nidogen-1 (NID1; UniProt P14543; 1247 aa) were retrieved from the UniProt database. The three-dimensional structures of NID1, the wild-type LAMC3 protein, and the mutant protein resulting from the c.1852_1882del p.(Pro618Serfs*5) variant were predicted using the AlphaFold Server (https://alphafoldserver.com, accessed on 19 June 2026). The mutant sequence was generated in silico by introducing the deletion at the coding level and translating the resulting open reading frame, yielding a truncated protein of 621 residues in which the native sequence diverges at codon 618 and terminates after four aberrant residues. Models were ranked by their predicted local distance difference test (pLDDT) and predicted TM-score (pTM) confidence metrics, and the top-ranked model was retained for downstream analysis.

To evaluate the effect of the truncation on partner binding, protein-to-protein docking was performed on the HDOCK server [13]. NID1 was selected as the binding partner based on the well-characterized laminin-to-nidogen interaction that anchors laminin networks within the basement membrane. The wild-type and mutant LAMC3 models were each docked as the receptor against the NID1 structure as the ligand. For each run, the resulting models were ranked by the HDOCK docking score and confidence score, and the top-ranked complex was retained. Interface residues of the receptor and ligand, together with their inter-residue contacts, were extracted from the HDOCK output using a 5 Å distance cutoff.

All predicted structures and docking complexes were visualized in PyMOL (v3.1.1). The wild-type and mutant models were superposed to illustrate the structural consequences of the truncation, and the interface residues at the LAMC3 to NID1 binding surface were mapped and rendered for both complexes.

### 2.3. Brain Tumor Molecular Analysis

Tumor molecular profiling was performed on formalin-fixed paraffin-embedded tissue from the surgically resected tectal lesion of Proband II.III. RNA and DNA were isolated using the Maxwell RSC RNA FFPE Kit and Maxwell RSC FFPE Plus DNA Kit (Promega, Madison, WI, USA), respectively. Targeted next-generation sequencing was performed using the Acibadem University Brain Tumor Research Group FusionPlex Brain Panel V1 (RNA) and VariantPlex Brain Panel V1 (DNA) on the NextSeq 500 Sequencing System (Illumina, San Diego, CA, USA). MGMT promoter methylation status was assessed by real-time PCR analysis.

### 2.4. AI Assistance Disclosure

During preparation of this manuscript, the authors used Claude Opus 4.8 (Anthropic) for language editing and grammar checking of the draft text; the tool was not used for data generation, analysis, or interpretation. All AI-assisted output was reviewed and verified by the authors, who take full responsibility for the content.

## 3. Results

A comprehensive review of the literature identified fewer than fifteen unrelated families with *LAMC3*-related disease reported to date, summarized together with the present cases in Table 1. Reported variants are predominantly protein-truncating (nonsense, frameshift, and canonical splice-site), consistent with a uniform loss-of-function mechanism, with rare missense changes occurring only in the compound heterozygous state. The associated phenotype is dominated by posterior cortical malformation, most commonly occipital pachygyria and polymicrogyria, accompanied by epilepsy of variable severity and largely preserved to mildly impaired cognition. White matter signal abnormalities have been described in only a minority of previously reported individuals, and consistently as a secondary or incidental feature, whereas intracranial neoplasia has never been reported. Against this background, the two present siblings, both homozygous for the novel c.1852_1882del p.(Pro618Serfs*5) variant, are distinguished as the first cases in which white matter disease constitutes the primary neuroimaging phenotype and the first to harbor an associated intracranial tumor (Table 1). For both siblings, whole-exome sequencing data were additionally reviewed for pathogenic or likely pathogenic variants in genes associated with established leukodystrophies and metabolic white matter disorders (including *PLP1*, *GFAP*, EIF2B1-5, *ASPA*, *GALC*, *ARSA*, and mitochondrial and peroxisomal disease genes), none of which were identified, further supporting *LAMC3* as the causative gene.

### 3.1. Clinical Findings

#### 3.1.1. Proband II.III

**Figure 1:**
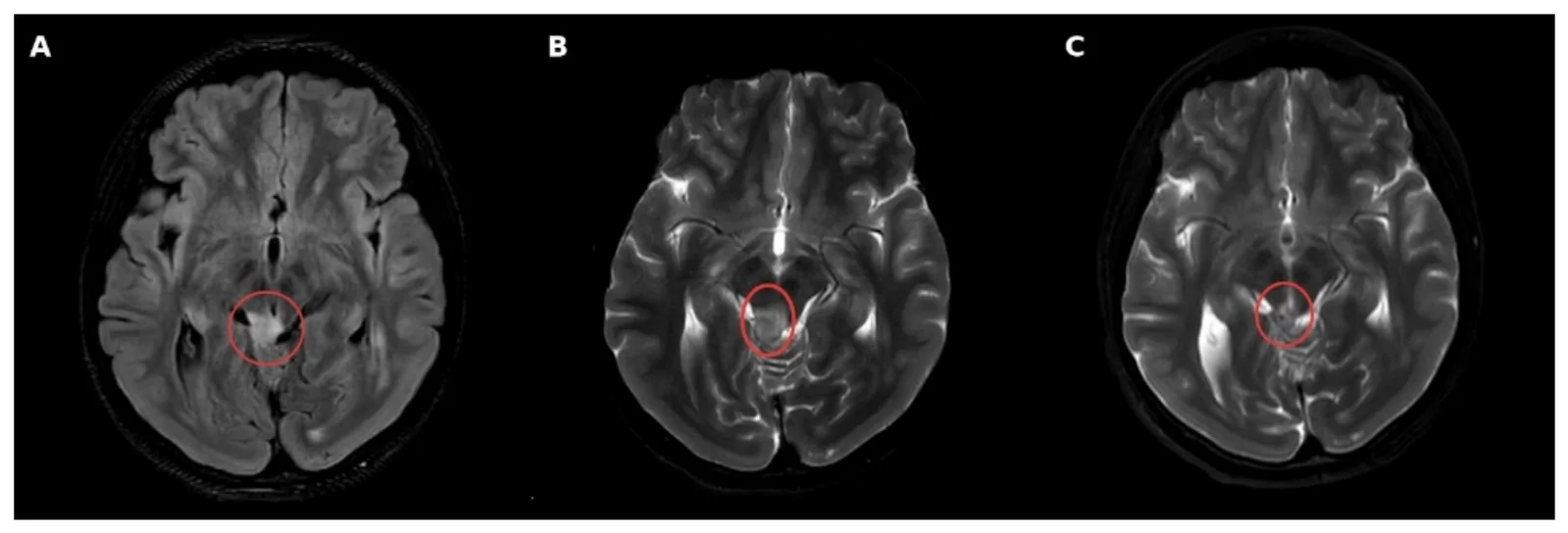
Brain Magnetic Resonance Imaging (MRI) features of the low-grade tectal glioneuronal tumor. (**A**) Axial Fluid-Attenuated Inversion Recovery (FLAIR) image demonstrates a well-circumscribed, hyperintense expansile lesion located in the tectal plate, with adjacent periventricular white matter signal change (red circle) (Pre-op). (**B**) Axial T2-weighted image shows an intrinsically hyperintense tectal mass (red circle) protruding into the quadrigeminal cistern, with narrowing of the cerebral aqueduct and no significant surrounding perilesional vasogenic edema (Pre-op). (**C**) Postoperative axial T2-weighted follow-up image depicts significant surgical debulking with a small, stable focus of residual tumor/postoperative changes along the tectal margin (red circle), and decompression of adjacent CSF pathways without significant surrounding edema.

A 17-year-old and 6-month-old female patient, born to second-degree consanguineous Turkish parents, presented with a history of epilepsy. Antenatal and perinatal histories were unremarkable, with no familial history of genetic, metabolic, or neurological diseases. Academic performance was comparable to peers. Following a syncopal episode, investigations revealed a pineal region mass lesion, for which the patient underwent two surgical interventions. Postoperative epileptic seizures were controlled with levetiracetam. Neurological examination revealed no intellectual disability. Visual field examination demonstrated a shift and visual blurring in the right eye, anatomically consistent with the lesion. Electroencephalography revealed 2–2.5 Hz sharp-slow wave paroxysms originating from the left central and bilateral temporal regions, showing secondary generalization lasting 20–30 s, accompanied by generalized fast wave discharges during non-REM sleep. Brain MRI demonstrated a nodular lesion in the right lateral half of the inferior mesencephalic tectum consistent with a presumed tectal glioma, with periventricular white matter gliotic T2/FLAIR hyperintense signal changes (Figure 1). Histopathological examination of the surgical excision material identified a low-grade glioneuronal tumor. Molecular analysis revealed a somatic *KRAS* (NM_004985.3) c.180_181delinsAA p.(Gln61Lys) pathogenic variant at VAF 42.9%, without pathogenic fusions, copy number variants, or MGMT promoter hypermethylation.

#### 3.1.2. Proband II.IV

**Figure 2:**
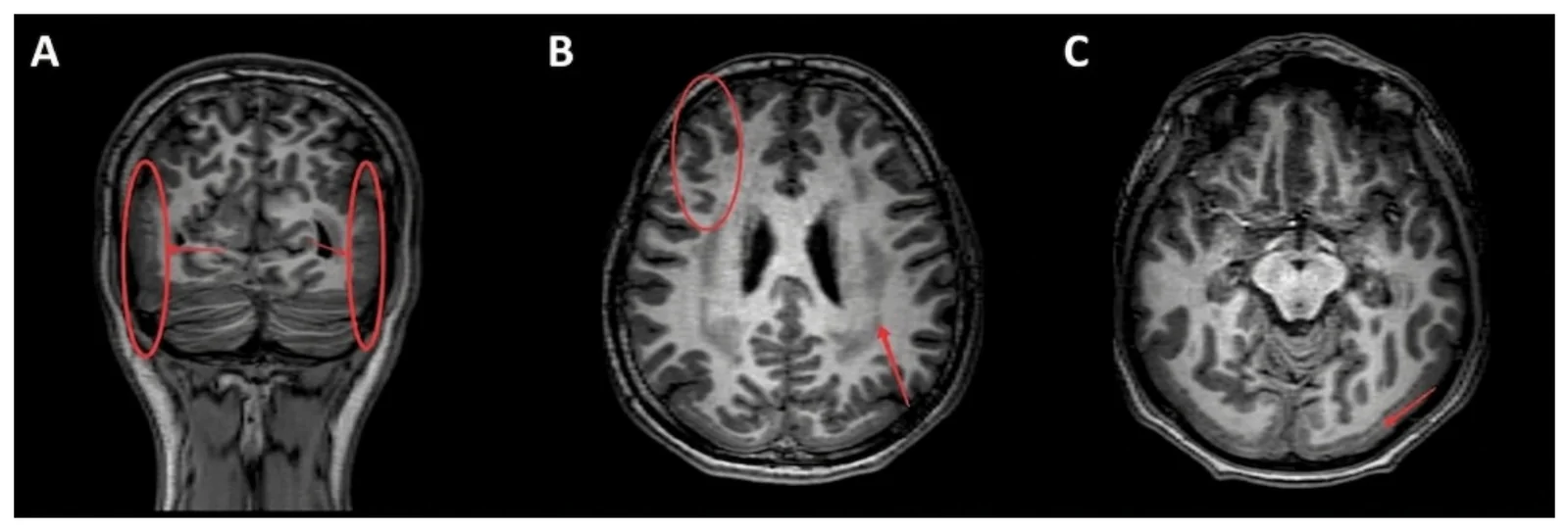
Cranial Magnetic Resonance Imaging (MRI) demonstrating complex malformations of cortical development, subcortical leukodystrophy-like changes, and subcortical U-fiber preservation. (**A**) Coronal T1-weighted image demonstrates bilateral symmetrical subcortical band heterotopia (red ovals) beneath the thickened cortex, accompanied by confluent subcortical white matter signal alterations mimicking a leukodystrophic pattern. (**B**) Axial T1-weighted image at the level of the lateral ventricles demonstrates an area of parietal polymicrogyria with irregular, closely packed gyri and shallow sulci overlying subcortical U-fibers that remain well-preserved (red circle), despite extensive adjacent leukodystrophy-like white matter abnormalities and posterior band heterotopia (red arrow). (**C**) Axial T1-weighted image at the posterior occipitotemporal level highlights marked posterior occipital pachygyria and a contiguous thick layer of heterotopic gray matter beneath the cortex (red arrow), confirming bilateral posterior-predominant band heterotopia (double cortex syndrome).

A 15-year-old and 4-month-old male patient, the sibling of Proband II.III, born of the same consanguineous union, presented with a history of epilepsy with onset at age 9 years. Antenatal and perinatal histories were unremarkable. Academic achievement was described as moderate. Seizures were controlled with carbamazepine. Neurological examination revealed mild cognitive deficit; cranial nerve examination, deep tendon reflexes, cerebellar tests, and visual field examination were normal. Electroencephalography demonstrated bilateral synchronous centrotemporal discharges, more prominent on the right side, absent during wakefulness and more pronounced during sleep. Brain MRI demonstrated bilateral periventricular deep white matter symmetric confluent T2/FLAIR hyperintense lesions at the level of the ventricular atria, without diffusion restriction, with relative sparing of U-fibers, basal ganglia, corticospinal tracts, and thalami, consistent with leukoencephalopathy (Figure 2). Additional findings included bilateral occipital pachygyria, parietal polymicrogyria, and subcortical band heterotopia. MR spectroscopy demonstrated mildly elevated Cho/Cr and Cho/NAA ratios consistent with ongoing demyelination and axonal injury. Cerebrospinal fluid analysis revealed an elevated protein level of 174 mg/dL with acellular cytology and normal glucose, chloride, and LDH values; CSF pressure was 18 cmH_2_O. Lysosomal enzyme panel was within normal limits, reducing the likelihood of a lysosomal storage disorder as the underlying etiology.

#### 3.1.3. Comparative Clinical Summary

Given the marked intrafamilial phenotypic discordance despite an identical homozygous genotype, the demographic, clinical, radiological, and molecular findings of the two siblings are summarized comparatively in Table 2. This side-by-side comparison highlights that, beyond their shared *LAMC3* genotype, the probands diverged across nearly every clinical domain, including the presence versus absence of intracranial neoplasia, the pattern and severity of white matter and cortical involvement, seizure semiology and pharmacological response, and cognitive outcome.

### 3.2. Genetic Findings

Whole-exome sequencing of the affected siblings identified a novel homozygous frameshift variant in *LAMC3* (NM_006059.4), c.1852_1882del; p.(Pro618Serfs*5), located in exon 13. This 31-base-pair deletion shifts the reading frame at codon 618, replacing proline with serine and introducing a premature termination codon after four aberrant residues. The variant is predicted to trigger nonsense-mediated mRNA decay or, alternatively, to yield a severely truncated protein of 621 residues that lacks approximately 61% of the 1575-residue wild-type chain, including the entire C-terminal region required for laminin network assembly. The variant was absent from population databases (gnomAD, 1000 Genomes) and had not been previously reported in ClinVar or HGMD.

**Figure 3:**
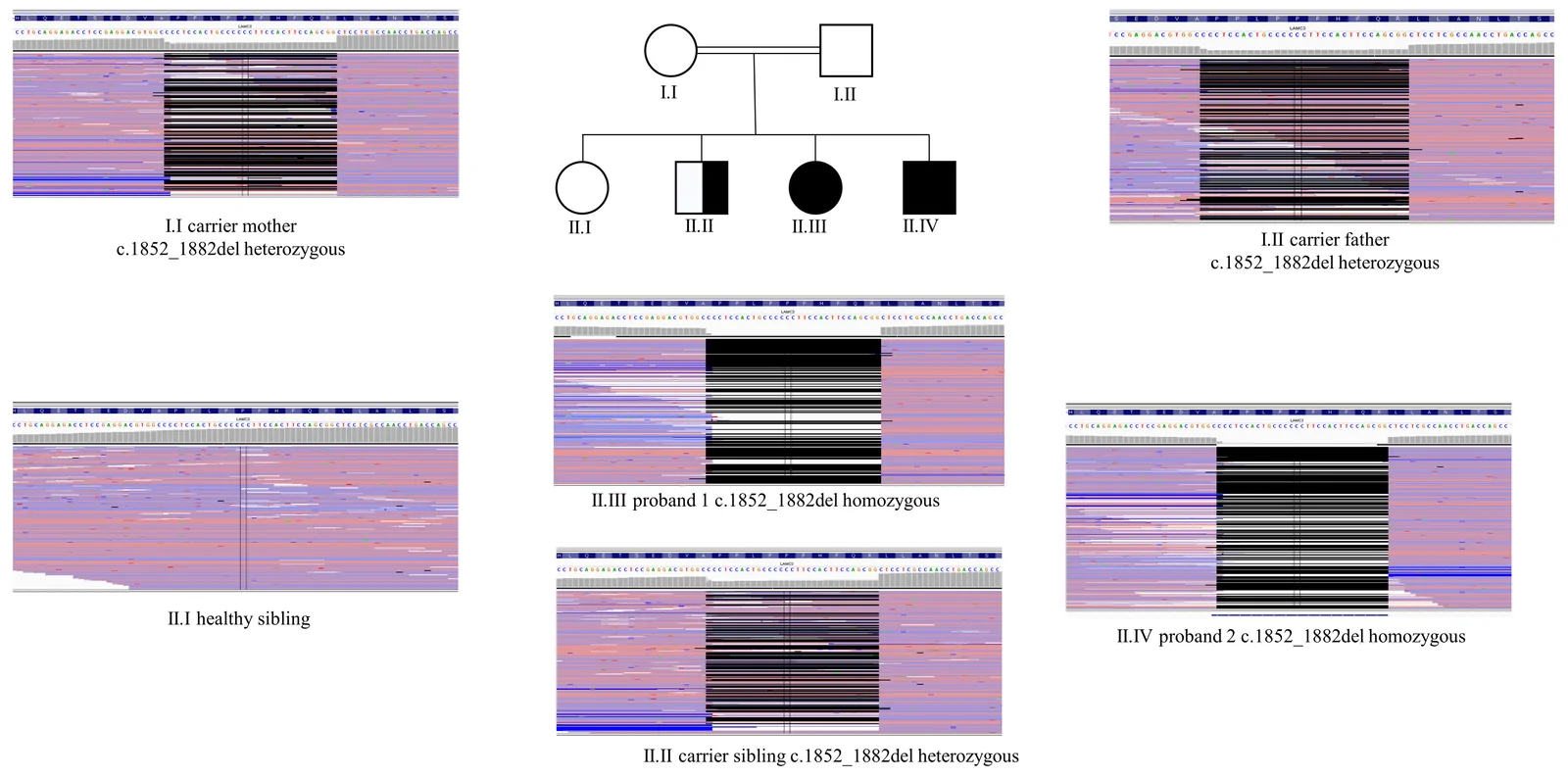
Pedigree and segregation of the *LAMC3* c.1852_1882del variant. The central pedigree depicts the consanguineous family; filled symbols denote the two affected siblings and the half-filled symbol denotes an unaffected heterozygous carrier. Surrounding panels show Integrative Genomics Viewer (IGV) read alignments across the variant region for each genotyped individual. Both parents (I.I, carrier mother; I.II, carrier father) and one unaffected sibling (II.II) are heterozygous for the 31 bp deletion; the two affected siblings (Proband II.III, Proband II.IV) are homozygous; and one unaffected sibling (II.I) does not carry the variant. The variant thus fully cosegregates with the disease phenotype in a manner consistent with autosomal recessive inheritance.

Segregation analysis was performed in all available family members by next-generation sequencing, with read alignments reviewed in the Integrative Genomics Viewer (IGV) to confirm zygosity in each individual Figure 3. The two affected siblings (Proband II.III and II.IV) were homozygous for the deletion, whereas both consanguineous parents were heterozygous carriers. Of the two unaffected siblings, one was a heterozygous carrier, and the other did not carry the variant. The variant therefore cosegregated fully with the disease phenotype in a manner consistent with autosomal recessive inheritance.

On the basis of these findings, the variant was classified as pathogenic according to the ACMG/AMP guidelines, meeting the criteria PVS1 (a null variant in a gene in which loss of function is an established disease mechanism, with the premature termination codon predicted to undergo nonsense-mediated decay), PM2 (absence from population databases), PP3 (computational prediction tools unanimously support a deleterious effect on the gene) and PP1 (cosegregation with disease in multiple affected family members).

### 3.3. Bioinformatic Findings

Multiple sequence alignment has revealed that the residues in and around Pro618 are highly conserved among vertebrate orthologs; this indicates that the affected region lies within a functionally restricted segment of the protein. The variant is predicted to affect the expression of the laminin/attractin EGF and laminin EGF domain regions located between residues 707–748 and 673–1010.

**Figure 4:**
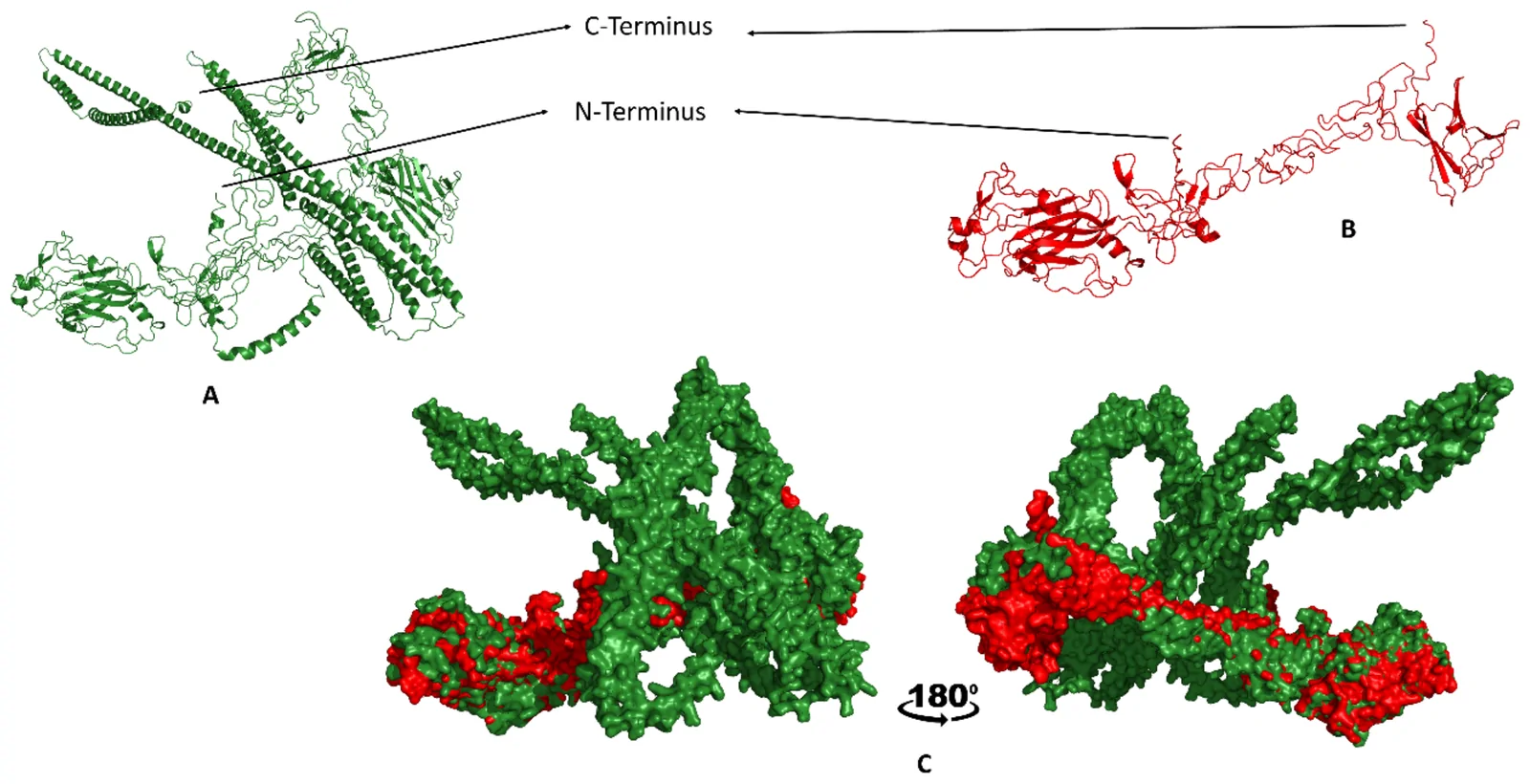
AlphaFold structural models of wild-type and mutant LAMC3. (**A**) Cartoon representation of the wild-type LAMC3 chain (green), showing the elongated multidomain laminin architecture, with the N-terminal and C-terminal regions indicated. (**B**) Cartoon representation of the mutant protein resulting from the c.1852_1882del (p.(Pro618Serfs*5)) variant (red), truncated to a compact 621-residue N-terminal fragment that lacks the entire C-terminal half of the chain. (**C**) Surface representation of the mutant model (red) superposed on the wild-type model (green), shown in two orientations related by a 180-degree rotation, illustrating that the retained region corresponds to only the N-terminal portion of the native chain.

AlphaFold modeling of the wild-type LAMC3 (pTM = 0.38) chain reproduced the expected elongated, multidomain laminin architecture, comprising a compact N-terminal globular region followed by an extended C-terminal arm (Figure 4A). The relatively low pTM score of the wild-type model (0.38) reflects reduced confidence in the relative inter-domain arrangement of this large, multidomain protein, and constitutes an additional limitation when interpreting the docking results derived from this model. In contrast, the model of the p.(Pro618Serfs*5) mutant (pTM = 0.73) was reduced to a compact N-terminal fragment of 621 residues that entirely lacked the C-terminal half of the chain, including the extended arm and the domains required for heterotrimer assembly and laminin network formation (Figure 4B). Superposition of the two models confirmed that the truncation removes approximately 61% of the native chain while leaving the retained N-terminal region essentially unchanged (Figure 4C). AlphaFold modeling of the wild-type LAMC3 protein yielded a full-length structure of 1575 residues with a mean per-residue pLDDT of 80.4, indicating overall reliable folding. The p.(Pro618Serfs*5) variant produced a severely truncated model of only 621 residues, consistent with premature termination shortly downstream of codon 618 and the loss of 954 C-terminal residues, corresponding to roughly 61% of the protein.

**Figure 5:**
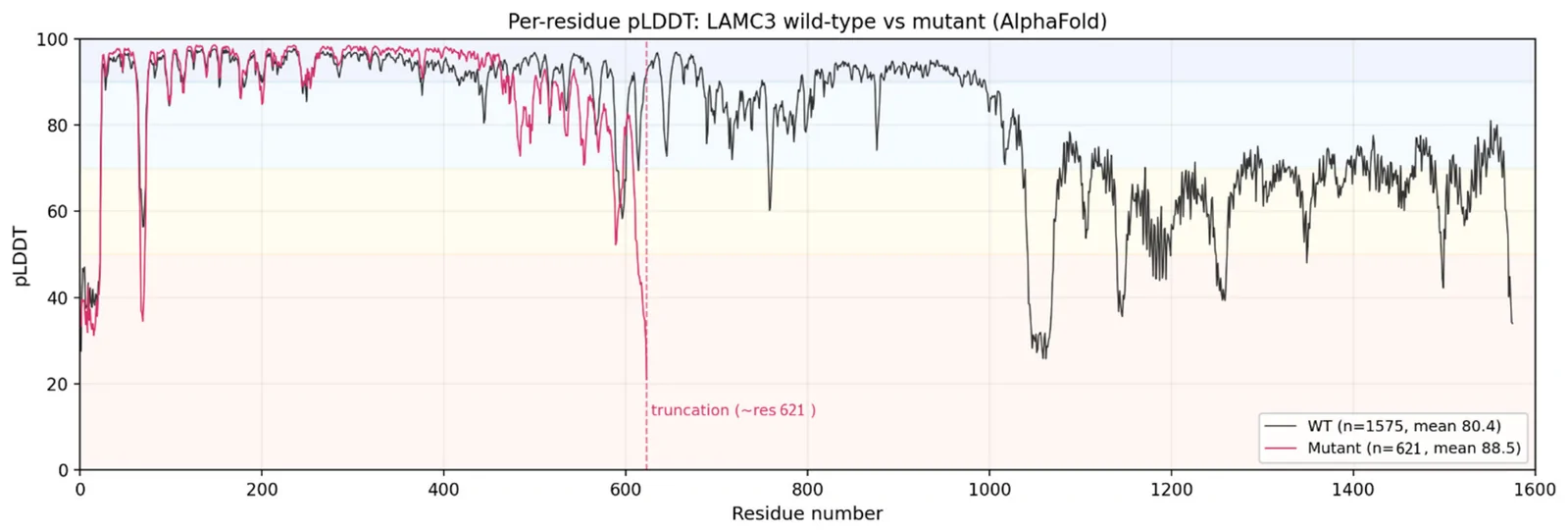
Per-residue pLDDT profiles of wild-type and mutant LAMC3 from AlphaFold modeling. The wild-type protein (1575 residues) is shown in black and the p.(Pro618Serfs*5) mutant (621 residues) in red; the dashed line marks the truncation point (The black lines show the pLDDT values for the wild-type protein and the red lines show those for the mutant protein).

This truncation eliminated the C-terminal region encompassing the laminin coiled-coil domain that mediates assembly of the γ3 chain into the laminin heterotrimer. Across the shared N-terminal segment (residues 1 to 621), both models folded with comparably high confidence (wild-type 90.4, mutant 88.6), confirming that the retained region is modeled reliably and that the structural divergence between the two is driven by truncation rather than local misfolding. The higher global mean pLDDT of the mutant model relative to the full-length wild-type (88.6 versus 80.4) reflects the removal of intrinsically low-confidence C-terminal domains and should not be interpreted as improved structural integrity (Figure 5).

**Figure 6:**
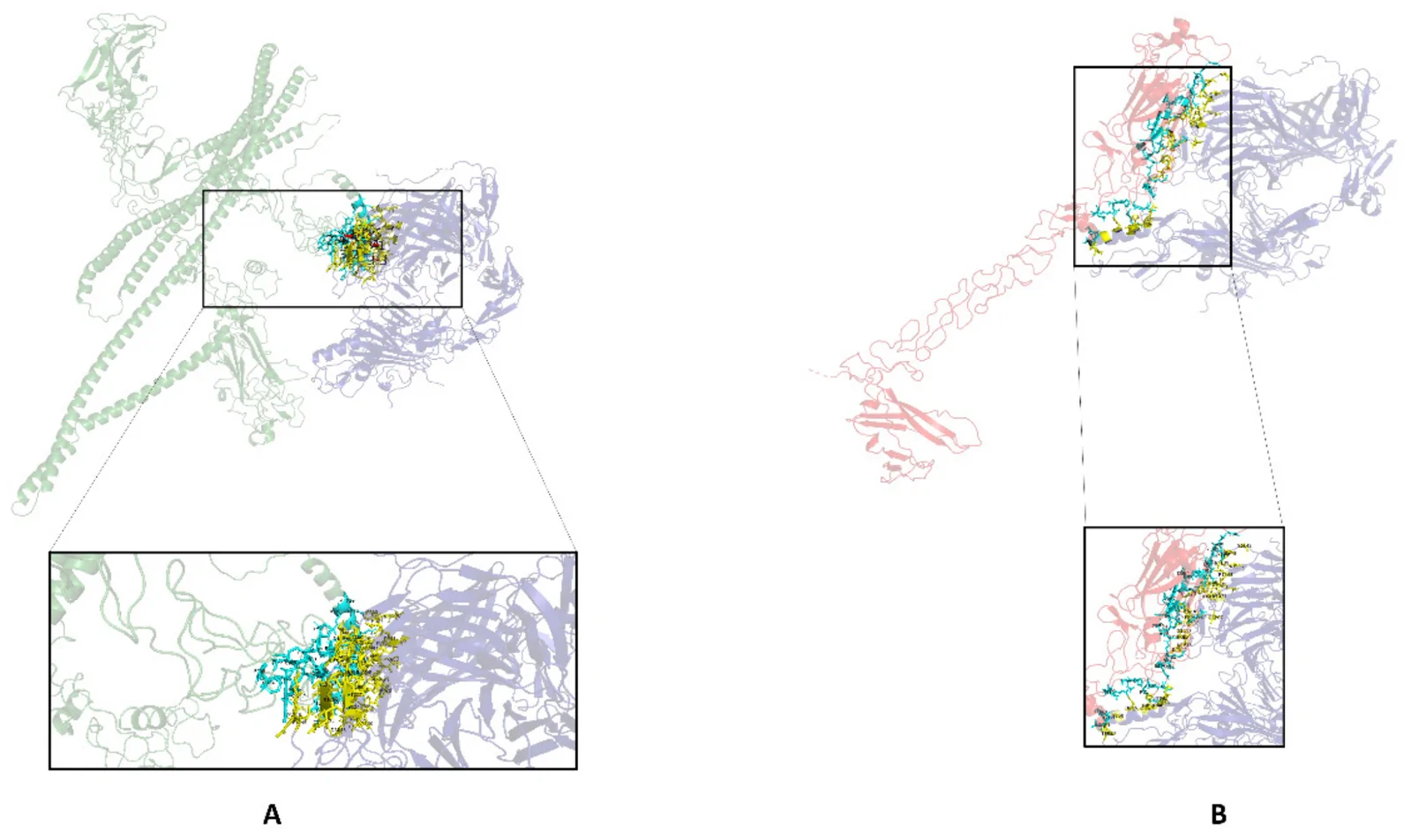
HDOCK docking of wild-type and mutant LAMC3 with nidogen-1 (NID1). (**A**) Top-ranked docking model of wild-type LAMC3 (green) with NID1 (blue); the boxed interface is enlarged below, with interface residues shown as sticks (LAMC3 in cyan, NID1 in yellow) and labeled. The interaction localizes to the C-terminal region of LAMC3 (residues 906 to 1029), corresponding to the physiological nidogen-binding region. (**B**) Top-ranked docking model of the mutant LAMC3 (red) with NID1 (blue); the boxed interface is enlarged. The truncation abolishes the native interface, and the residual interaction relocates to a non-native N-terminal surface of the truncated chain (residues 16 to 348) (The dashed lines indicate the enlarged areas of interaction regions in protein docking. Frames are a zoomed-in view of the interaction area.).

To assess the impact of the truncation on the interaction with nidogen-1, the wild-type and mutant LAMC3 models were each docked against NID1 using HDOCK (Figure 6, Table 3). Both complexes yielded high-confidence top-ranked models. In the wild-type complex, the LAMC3 interface localized to the C-terminal region of the chain (residues 906 to 1029), engaging NID1 through 114 inter-residue contacts. This region falls within the laminin EGF-like (LE) domain segment that, in laminin gamma chains, harbors the canonical nidogen-binding module, consistent with a physiologically relevant interface.

In the mutant complex, this native interface was necessarily absent, as the truncation terminates the protein at residue 621, well upstream of residues 906 to 1029. Consequently, docking engaged an entirely different, N-terminal surface of the truncated chain (residues 16 to 348), corresponding to the laminin N-terminal (LN) domain region, through 105 contacts. Although the mutant complex returned a nominally more favorable docking score (−338.96 versus −308.37) and confidence score (0.98 versus 0.96), these values reflect the shape and energetic complementarity of the top-ranked pose rather than physiological binding affinity or biological validity. The relocation of the interface from the native C-terminal nidogen-binding region to a non-native N-terminal surface indicates that the truncated protein cannot reconstitute the physiological laminin-to-nidogen interface, and that any residual interaction is aberrant and unlikely to support functional basement membrane assembly. These docking-based inferences are computational and hypothesis-generating; direct experimental confirmation (e.g., co-immunoprecipitation, surface plasmon resonance, or isothermal titration calorimetry) will be required to establish definitive loss of physiological binding.

## 4. Discussion

The loss-of-function consequence predicted for the c.1852_1882del p.(Pro618Serfs*5) variant is supported by convergent genetic, clinical, and computational evidence, and is further consistent with previously published experimental studies on the laminin-nidogen interaction and LAMC3 ablation in mice. Frameshift variants that introduce a premature termination codon upstream of the final exon–exon junction are efficiently recognized by the nonsense-mediated mRNA decay (NMD) pathway, which degrades the aberrant transcript and precludes synthesis of the truncated protein [14]. Genome-wide bioinformatic modeling of these NMD rules has confirmed that such protein-truncating variants predominantly act through loss of function across human disease genes [15]. The present variant lies in exon 13, well upstream of the final exon junction, and is therefore predicted to elicit NMD. Even in the alternative scenario of NMD escape, the resulting protein would lack the entire C-terminal half of the chain, including the laminin EGF-like (LE) modules that harbor the nidogen-binding site experimentally defined by crystallography of the laminin-to-nidogen complex [16] consistent with this, our wild-type docking localized LAMC3 to the NID1 interface to residues 906 to 1029, a region entirely absent from the truncated mutant. Both outcomes therefore converge on abolition of laminin gamma-3 function, in agreement with experimental ablation of LAMC3 in mice, which disrupts cortical basement membrane integrity and lamination [17], and with the exclusively loss-of-function mutational spectrum reported for *LAMC3*-related disease.

Premature truncation is a recurrent mechanism in *LAMC3*-related disease. Barak et al. first established recessive loss-of-function *LAMC3* variants as a cause of occipital cortical malformation in consanguineous families ascertained through first-cousin marriage, subsequently identified across three unrelated Turkish families with occipital polymicrogyria and epilepsy [3]. Later reports repeatedly implicated nonsense and frameshift variants that cause premature truncation of the laminin γ3 chain, and the associated phenotype has progressively broadened beyond the occipital lobe, including a novel stop-gain variant (p.Arg1291*) with polymicrogyria extending into temporal regions and biallelic variants with predominantly temporal rather than occipital involvement. The variant reported here terminates the protein considerably earlier than most previously described alleles, removing the entire C-terminal half, including the assembly-critical coiled-coil domain, which provides a plausible structural basis for complete loss of function [4,10,11]. Our analysis also underscores an interpretive caveat for structural studies of truncating variants: AlphaFold confidence scores such as pLDDT and pTM are length-dependent, and a truncated model may return a higher global mean simply because the disordered, low-confidence C-terminal regions of the full-length protein are no longer present. Confidence metrics should therefore be compared over the shared sequence rather than globally, as done here, to avoid mistaking the loss of protein for an improvement in model quality.

*LAMC3*-associated cortical malformations represent an exceptionally rare autosomal recessive condition caused by biallelic loss-of-function variants in the gene encoding the laminin γ3 chain, a critical component of the extracellular matrix. Since their initial description by [3] in consanguineous Turkish families presenting with complex bilateral occipital gyration abnormalities, fewer than fifteen unrelated families have been reported in the global literature. *LAMC3* is located on chromosome 9q34.12 and encodes a 1575-amino acid protein that assembles into heterotrimeric laminin complexes through non-covalent interactions with laminin α and β chains [5,10]. These laminin isoforms are essential components of basement membranes and play indispensable roles in cell adhesion, differentiation, migration, and extracellular matrix organization throughout development [18]. In the developing human fetal brain, *LAMC3* expression peaks between late gestation and late infancy, localizing to the cell bodies and apical dendrites of pyramidal neurons in the cortical plate [3].

The phenotypic spectrum of *LAMC3*-related disease has expanded considerably since its initial description. Barak et al. established bilateral occipital pachygyria and polymicrogyria as the defining phenotype [3]. Zambonin et al. reported generalized polymicrogyria extending to the frontal, parietal, and temporal cortices with posterior deep white matter signal changes [5]. Kasper et al. described frontal-only polymicrogyria with adult-onset epilepsy, demonstrating that occipital involvement is not obligatory [9]. De Angelis et al. reported posterior periventricular nodular heterotopia [6]; Abe et al. described temporal cobblestone malformation with ventral pontine involvement [11]; and Falcicchio et al. reported drug-resistant epilepsy with pontocerebellar atrophy [4]. Most recently, Saarela et al. described a broad phenotypic spectrum ranging from Lennox–Gastaut syndrome to focal epilepsy with preserved cognition [12]. Against this background of progressive phenotypic expansion, the probands add two entirely novel clinical dimensions: intracranial neoplasia and primary leukoencephalopathy.

The first major novel finding of the present study is the low-grade glioneuronal tumor identified in the tectal region of Proband II.III. Histopathological examination identified spindle cell morphology consistent with a low-grade glioneuronal tumor; molecular analysis revealed a somatic *KRAS* c.180_181delinsAA p.(Gln61Lys) pathogenic variant at VAF 42.9%. Tectal gliomas are known to harbor high rates of activating *KRAS* mutations [19]; rare *KRAS* missense variants at codon 61 have been previously described [20,21]. The co-occurrence of *LAMC3*-associated cortical malformation with intracranial neoplasia has not been previously reported in the literature. It is conceivable that germline loss-of-function of *LAMC3* may disrupt the integrity of the extracellular matrix microenvironment surrounding glial progenitors, potentially predisposing to somatic oncogenic events such as activating *KRAS* [10,22]. This hypothesis is speculative and based on a single observation; a coincidental co-occurrence of a sporadic KRAS driver mutation, unrelated to the germline *LAMC3* variant, cannot be excluded. Systematic evaluation of tumor occurrence in additional *LAMC3*-related disease cohorts will be necessary to determine whether this represents a genuine biological association. Furthermore, the temporal relationship between the patient’s multiple surgical interventions and the white matter signal abnormalities documented on follow-up imaging raises the possibility that a proportion of the white matter signal abnormalities may have been treatment-related. Notably, preoperative FLAIR imaging (Figure 1A) already demonstrated periventricular white matter signal change prior to surgical intervention, indicating that this finding, at least in part, reflects an underlying process related to the LAMC3 variant rather than being purely a postoperative artifact, although a treatment-related contribution to signal evolution on subsequent follow-up imaging cannot be entirely excluded.

The dominant neuroimaging finding in Proband II.IV was confluent leukoencephalopathy with symmetric, merging T2/FLAIR hyperintense lesions bilaterally involving the corona radiata, centrum semiovale, and peritrigonal white matter, with relative sparing of U-fibers, basal ganglia, corticospinal tracts, and thalami, and without diffusion restriction. This pattern initially raised the consideration of leukodystrophic conditions—most notably metachromatic leukodystrophy, given the symmetric posterior predominance, the patient’s age, and the absence of diffusion restriction—underscoring the diagnostic challenge posed by white matter-predominant presentations of *LAMC3*-related disease. Comprehensive metabolic workup, including lysosomal enzyme panel analysis, returned within normal limits; whole-exome sequencing data were further reviewed for pathogenic variants in nuclear genes associated with mitochondrial and peroxisomal disorders, none of which were identified, reducing the likelihood of these alternative metabolic etiologies. However, functional biochemical testing and evaluation of mitochondrial DNA-encoded genes were not performed in this study and cannot be formally excluded by sequencing alone. Cerebrospinal fluid analysis demonstrated an elevated protein level of 174 mg/dL with acellular cytology and normal glucose, chloride, and LDH values; CSF pressure was 18 cmH_2_O. While CSF protein elevation is a non-specific finding, it is consistent with an active white matter process and has been described in other genetic leukoencephalopathies. MR spectroscopy demonstrated mildly elevated Cho/Cr and Cho/NAA ratios without tumor-specific alterations, interpreted as consistent with ongoing demyelination and axonal injury, further supporting an active rather than purely static white matter pathology. Taken together, these findings suggest that the leukoencephalopathy in this patient may harbor a progressive or dynamic component, meriting longitudinal neuroimaging and biochemical surveillance. The genetic etiology was ultimately confirmed by identification of the homozygous *LAMC3* frameshift variant, highlighting the importance of whole-exome sequencing in the diagnostic workup of pediatric leukoencephalopathies of undetermined etiology, particularly in the setting of consanguinity. Proband II.III demonstrated periventricular white matter gliosis without overt leukoencephalopathy, representing a milder end of the same white matter pathology spectrum. Although white matter signal abnormalities have been reported as incidental findings in prior *LAMC3* reports [5,12], the probands are the first in which they constitute the primary neuroimaging phenotype. Demirayak et al. documented marked microstructural disruption across multiple major white matter tracts in a patient with homozygous *LAMC3* [23], while Urgen et al. and Roberto et al. demonstrated that structural abnormalities in *LAMC3* carriers extend well beyond the occipital cortex [20,24].

From a genetic standpoint, the c.1852_1882del p(.Pro618Serfs*5) variant represents a novel 31-base-pair deletion in exon 13 of *LAMC3*, predicted to result in loss of function through protein truncation or nonsense-mediated mRNA decay. The variant is absent from population databases (gnomAD, ClinVar, HGMD) and was classified as pathogenic (PVS1, PM2, PP3, PP1) under the ACMG/AMP framework [7]. The marked phenotypic discordance between the two homozygous siblings—Proband II.III presenting with tectal glioma and periventricular white matter gliosis, Proband II.IV with confluent leukoencephalopathy, occipital pachygyria, parietal polymicrogyria, and subcortical band heterotopia—represents a striking example of intrafamilial phenotypic variability in *LAMC3*-related disease. Similar variability has been previously described by Afawi et al. and Saarela et al. [8,12]. The mechanisms underlying this intrafamilial variability remain incompletely understood but likely involve modifier gene effects, stochastic variation in developmental processes, epigenetic regulation of *LAMC3* expression, and the influence of somatic mutational events on the ultimate clinical phenotype.

## 5. Conclusions

In conclusion, the present study expands the phenotypic spectrum of *LAMC3*-related disease to include primary leukoencephalopathy, and reports the co-occurrence of a low-grade tectal glioneuronal tumor as a novel, single-case observation whose causal relationship to the *LAMC3* variant remains unproven and warrants further study, in two siblings harboring a novel homozygous frameshift variant. *LAMC3* should be considered in the differential diagnosis of pediatric leukoencephalopathies of uncertain etiology, particularly in the presence of consanguinity. Accumulation of additional cases through international collaborative registries will be essential to elucidate the relationship between germline *LAMC3* loss-of-function and intracranial neoplasia, and to fully delineate the phenotypic spectrum of this rare disorder.

## Figures and Tables

**Table 1:** Summary of all reported *LAMC3*-associated cases including present cases.

Patient	Sex	Age	Seizure Onset	Ethnicity	Variant	Variant Type	Epilepsy Syndrome	MRI Findings	White Matter	Tumor	Cognition	ACMG Criterias
Barak et al. [3]	F	14 y	2 y	Turkish	c.903_904del p.(Cys301*)	Frameshift [homozygous]	Generalized epilepsy	Bilateral occipital pachygyria and parieto-occipital PMG	No	No	Mild developmental delay	P(PM3,PVS1,PM2,PP5)
Barak et al. [3]	F	33 y	10 y	Turkish	c.470G>A p.(Trp157*)	Nonsense [homozygous]	Focal epilepsy	Bilateral occipital pachygyria and polymicrogyria	No	No	Normal	P(PVS1,PM2,PP5)
Barak et al. [3]	F	16 y	10 y	Turkish	c.1156C>T p.(Gln386*) + c.1048G>A p.(Gly350Arg)	Nonsense + Missense (comp. het.)	Focal epilepsy	Bilateral occipital pachygyria and polymicrogyria	No	No	Unknown	P(PVS1,PM2,PP5) VUS(PM2,PP3)
Afawi et al. [8]	M/F (×7)	NA	NA	Arab-Israeli	c.4086delA p.(Asp1363Thrfs*54)	Frameshift [homozygous]	Epilepsy with myoclonic-atonic seizures	Occipital malformation (CT unremarkable in most)	No	No	Borderline to moderate developmental delay	LP(PVS1,PM2)
Zambonin et al. [5]	F	14 y	3 y	Pakistani-Canadian	c.3190C>T p.(Gln1064*)	Nonsense [homozygous]	Epilepsy with myoclonic-atonic seizures	Bilateral frontal, parietal, temporal, occipital PMG; occipital pachygyria	Yes (posterior deep WM changes)	No	Severe developmental delay	LP(PVS1,PM2)
Kasper et al. [9]	F	30 y	30 y	German	c.976+1G>C	Splice site [homozygous]	Focal epilepsy (adult-onset)	Bilateral frontal PMG only (occipital spared)	No	No	Normal; frontal lobe dysfunction	P(PM3,PVS1,PM2)
De Angelis et al. [6]	M (fetus)	21 wk	NA	Australian	c.1066C>T p.(Arg356Cys) + c.2726A>G p.(Gln909Arg)	Missense + Missense (comp. het.)	NA (fetus)	Posterior periventricular nodular heterotopia (occipital horns)	No	No	NA	VUS(PM2,BP6)
Qian et al. [10]	F	24 y	13 y	Chinese	c.470G>A p.(Trp157*) + c.4030+1G>A	Nonsense + Splice (comp. het.)	Generalized epilepsy	Normal brain MRI	No	No	Normal development	P(PVS1,PM2,PP5) LP(PVS1,PM2)
Abe et al. [11]	M	24 y	8 y	Japanese	c.976+1G>A + c.4102_4105del p.(Arg1368Serfs*48)	Splice + Frameshift (comp. het.)	Focal epilepsy	Bilateral temporal cobblestone malformation; mild pontine midline malformation	No	No	Mild developmental delay (IQ: 80)	P(PM3,PVS1,PM2) LP(PVS1,PM2)
Falcicchio et al. [4]	M	28 y	6 y	Italian	c.3871C>T p.(Arg1291*)	Nonsense [homozygous]	Drug-resistant epilepsy (DEE)	Bilateral temporal and occipital PMG; cerebellum/pons atrophy	No	No	Moderate developmental delay (IQ: 48)	P(PM3,PVS1,PM2)
Saarela et al. [12] (P1)	M	11 y	1 y	Finnish	c.1866del p.(Phe623Serfs*10)	Frameshift [homozygous]	Lennox–Gastaut syndrome	Bilateral pachygyria-agyria + PMG; periventricular WM hyperintensity + leukomalacia	Yes (periventricular WM hyperintensity + leukomalacia)	No	Mild ID; autistic features; poor vision	VUS(PVS1)
Saarela et al. [12] (P2)	F	14 y	1 y	Finnish	c.1866del p.(Phe623Serfs*10)	Frameshift [homozygous]	Lennox–Gastaut syndrome	Bilateral pachygyria-agyria + PMG; periventricular WM hyperintensity	Yes (periventricular WM hyperintensity)	No	Mild ID; scoliosis	VUS(PVS1)
Saarela et al. [12] (P3)	M	35 y	8 y	Finnish	c.1866del p.(Phe623Serfs*10)	Frameshift [homozygous]	Focal epilepsy	Bilateral occipito-temporal PMG; periventricular WM hyperintensity	Yes (periventricular WM hyperintensity)	No	Normal cognition	VUS(PVS1)
Saarela et al. [12] (P4)	F	51 y	10 y	Finnish	c.1866del p.(Phe623Serfs*10) + c.4231-12C>G	Frameshift + Intronic [comp. het.]	Focal epilepsy	Large left temporo-parieto-occipital PMG + left occipital pachygyria; right frontoparietal PMG	No	No	Normal cognition; severe migraine	VUS(PVS1) VUS(PM2)
Proband II.III	F	17 y 6 m	Postoperative	Turkish	c.1852_1882del p.(Pro618Serfs*5)	Frameshift [homozygous, novel]	Focal epilepsy (postoperative)	Tectal low-grade glioneuronal tumor; periventricular white matter gliotic changes	Yes (periventricular gliosis)	Yes (tectal glioma, KRAS p.(Gln61Lys)	Normal	P(PVS1, PM2, PP3, PP1)
Proband II.IV	M	15 y 4 m	9 y	Turkish	c.1852_1882del p.(Pro618Serfs*5)	Frameshift [homozygous, novel]	Focal epilepsy	Confluent leukoencephalopathy (corona radiata, centrum semiovale, peritrigonal WM); bilateral occipital pachygyria, parietal PMG, subcortical band heterotopia	Yes (confluent leukoencephalopathy)	No	Mild cognitive deficit	P(PVS1, PM2, PP3, PP1)

ACMG: American College of Medical Genetics and Genomics; LP: likely pathogenic; P: pathogenic; PMG: polymicrogyria; WM: white matter; ID: intellectual disability; TC: tonic–clonic; DEE: developmental and epileptic encephalopathy; comp. het.: compound heterozygous; VUS: variant of uncertain significance; NA: not available.

**Table 2:** Comparative clinical summary of the two *LAMC3* probands.

Category	Parameter	Proband II.III (Female)	Proband II.IV (Male)
1. Demographic and General Information			
Age (at time of evaluation)		17 years 6 months	15 years 4 months
Sex		Female	Male
Variant	Genotype	Homozygous *LAMC3* (NM_006059.4) c.1852_1882del	Homozygous LAMC3 (NM_006059.4) c.1852_1882del
2. Neurological Findings and Symptoms			
Brain tumor	Type/grade	Low-grade tectal glioneuronal tumor (spindle cell morphology)	None
Somatic tumor variant	Gene/HGVS notation	*KRAS* (NM_004985.3) c.180_181delinsAA p.(Gln61Lys), VAF 42.9%	Not applicable
Tumor molecular profile	Fusions/CNV/MGMT	No pathogenic fusions or copy number variants; MGMT promoter unmethylated	Not applicable
Surgical history	Interventions	Two surgical resections (pineal region mass)	None
White matter disease	Leukodystrophy/demyelination	Periventricular gliosis (mild, secondary finding)	Confluent leukoencephalopathy (primary neuroimaging finding)
Cortical malformation	Pachygyria/polymicrogyria	*Not reported*	Bilateral occipital pachygyria; parietal polymicrogyria; subcortical band heterotopia
Epilepsy/seizures	Type and onset	Postoperative focal epilepsy; controlled with levetiracetam	Onset at age 9 years; focal epilepsy; controlled with carbamazepine
Intellectual disability	Neuropsychological assessment	None; no intellectual disability	Mild cognitive deficit
Neurological examination	Clinical findings	Right-sided visual field shift/blurring (lesion-related)	Cranial nerves, DTRs, cerebellar testing, visual fields: normal
EEG	Findings	2–2.5 Hz sharp-slow wave paroxysms, left central and bilateral temporal, secondary generalization (20–30 s), generalized fast waves in NREM sleep	Bilateral synchronous centrotemporal discharges, right-predominant; absent in wakefulness, prominent in sleep
Academic performance		Comparable to peers	Moderate
3. Radiological Evaluation			
Brain MRI	*T1/T2/FLAIR/DWI*	Tectal nodular lesion; periventricular T2/FLAIR hyperintensity (Figure 1)	Bilateral confluent periventricular T2/FLAIR hyperintensity; no diffusion restriction; U-fibers, basal ganglia, corticospinal tracts, thalami spared (Figure 2)
MR Spectroscopy	*Metabolite profile*	*Not performed/not reported*	Mildly elevated Cho/Cr and Cho/NAA ratios, consistent with demyelination/axonal injury

Cho/Cr: choline-to-creatine ratio; Cho/NAA: choline-to-N-acetylaspartate ratio; CNV: copy number variant; DTR: deep tendon reflex; DWI: diffusion-weighted imaging; EEG: electroencephalography; FLAIR: fluid-attenuated inversion recovery; HGVS: Human Genome Variation Society; MGMT: O6-methylguanine-DNA methyltransferase; MRI: magnetic resonance imaging; NREM: non-rapid eye movement; T1: T1-weighted imaging; T2: T2-weighted imaging; VAF: variant allele frequency.

**Table 3:** HDOCK docking of wild-type and mutant LAMC3 with nidogen-1 (NID1), top-ranked model.

Parameter	Wild LAMC3 + NID1	Mutant LAMC3 + NID1
**Docking score**	−308.37	−338.96
**Confidence score**	0.96	0.98
**Ligand RMSD (Å)**	102.58	96.41
**Inter-residue contacts (n)**	114	105
**LAMC3 interface residues (n)**	44	50
**LAMC3 interface region**	residues 906-1029 (C-terminal LE/nidogen-binding region)	residues 16-348 (N-terminal LN region)
**NID1 interface region**	residues 229-1247 (G2 and G3 domains)	residues 833-1242 (predominantly G3)
**Interface localization**	Native	Non-native (native region deleted)

Note: The mutant docking score reflects algorithmic shape and energetic complementarity at a non-native N-terminal surface and does not represent physiological binding affinity; the native nidogen-binding interface (residues 906–1029) is absent in the truncated protein.

## Data Availability

The original contributions presented in this study are included in the article material. Further inquiries can be directed to the corresponding author.
